# GPR171 Activation Modulates Nociceptor Functions, Alleviating Pathologic Pain

**DOI:** 10.3390/biomedicines9030256

**Published:** 2021-03-05

**Authors:** Pyung Sun Cho, Han Kyu Lee, Young In Choi, Seung In Choi, Ji Yeon Lim, Minseok Kim, Hyun Kim, Sung Jun Jung, Sun Wook Hwang

**Affiliations:** 1 Department of Biomedical Sciences, College of Medicine, Korea University, Seoul 02841, Korea; physicho0@korea.ac.kr (P.S.C.); csiat@korea.ac.kr (S.I.C.); ljyangel1004@korea.ac.kr (J.Y.L.); minseok7929@korea.ac.kr (M.K.); 2 Department of Physiology, College of Medicine, Korea University, Seoul 02841, Korea; 3 Department of Physiology, College of Medicine, Hanyang University, Seoul 04763, Korea; zaguar@hanyang.ac.kr (H.K.L.); cyi2012@naver.com (Y.I.C.); 4 Department of Anatomy, College of Medicine, Korea University, Seoul 02841, Korea; kimhyun@korea.ac.kr

**Keywords:** GPR171, pain, analgesia, nociceptor, TRP channel

## Abstract

Modulation of the function of somatosensory neurons is an important analgesic strategy, requiring the proposal of novel molecular targets. Many G-protein-coupled receptors (GPRs) have been deorphanized, but the receptor locations, outcomes due to their activations, and their signal transductions remain to be elucidated, regarding the somatosensory nociceptor function. Here we report that GPR171, expressed in a nociceptor subpopulation, attenuated pain signals via Gi/o-coupled modulation of the activities of nociceptive ion channels when activated by its newly found ligands. Administration of its natural peptide ligand and a synthetic chemical ligand alleviated nociceptor-mediated acute pain aggravations and also relieved pathologic pain at nanomolar and micromolar ranges. This study suggests that functional alteration of the nociceptor neurons by GPR171 signaling results in pain alleviation and indicates that GPR171 is a promising molecular target for peripheral pain modulation.

## 1. Introduction

Nociceptor neurons in the dorsal root ganglia (DRG) are in charge of the initiation of pain-related signals in the body [1]. Upon activation by ligands, G-protein-coupled receptors (GPRs) modulate the excitability of nociceptors, negatively or positively tuning the strength of the initiated signals. While some GPRs, such as prostaglandin EP2 and EP4 receptors and bradykinin B2 receptors strongly facilitate nociceptor excitation, other GPRs, including μ-opioid receptors, GABA_B_ receptors, and chemokine-like receptor 1 receptors, reduce excitation, highly contributing to relieving pain [2,3,4,5,6]. Better understanding of the identities of pain-relieving GPRs and the molecular mechanisms underlying the reduction in excitability may help to contribute to the development of novel painkilling strategies. GPR171 was initially cloned two decades ago but was recently deorphanized [7,8]. Here we examined its expression in the nociceptor population and hypothesized that GPR171 activation by its specific ligands may modulate nociceptor functions, consequently regulating pain phenotypes.

## 2. Materials and Methods

### 2.1. Nociceptive Behavioral Tests

Six-week-old male C57BL/6J mice were used. Time engaged in hind paw licking and flicking behavior was quantitated for ~10 min as previously described [9,10,11] (Supplementary Figure S8A). For eliciting TRPV1-activation-induced behaviors, 100 ng capsaicin-containing 10 μL saline was intraplantarily injected; for eliciting TRPA1-activation-induced behaviors, 100 ng AITC in 10 μL saline or 26.4 μg cinnamaldehyde in 20 μL saline was injected. For eliciting depolarization-induced behaviors mediated by voltage-gated channel activation, 140 mM KCl in 10 μL water was injected. For conventional TRPV4-mediated flinch assays, we used intraplantar injection of 10 μL deionized water for hypotonic stimulation 30 min after prostaglandin E2 (PGE2) priming (intraplantar pretreatment with 10 μL saline containing 100 ng PGE2) [11]. TRPV4-mediated nociceptive behaviors were observed by counting the number of hind paw flinching events for 10 min immediately after the hypotonic stimulus. For observing formalin-induced acute and tonic pain, 5% or 0.5% formalin in saline (20 μL) was intraplantarily injected and the cumulative time that a mouse spent licking and flinching the injected paw was measured every 5 min for 50 min.

For CFA-induced inflammation, 10 μL CFA was injected into a hind paw (Supplementary Figure S8D). For determining changes in mechanical or thermal behaviors by 24 and 48 h CFA inflammation, mice were acclimated to the test environment for 30 min before performing the following assays. Hargreaves assay (using a Plantar Analgesia meter; IITC, Woodland Hills, CA, USA) for acute heat avoidance or thermal hyperalgesia, von Frey assay by up-and-down paradigm (using von Frey filaments; Stoelting, Dale Wood, IL, USA) for mechanical allodynia, and Randall–Selitto assay (using Analgesy-meter, UGO Basile, Italy) for flexion reflex to noxious pressures, were carried out as described previously [12,13,14,15,16] (Supplementary Figure S8C).

For neuropathic pain development, the CCI model was established as described previously [17] (Supplementary Figure S8D). Briefly, the mice were anesthetized with inhalation of 3% isoflurane in a mixture of N_2_O/O_2_ gas. The full circumference of the sciatic nerve of the left hind limb was exposed and loosely tied three times with two silk sutures (7-0; Ailee, Busan, Korea). Sham surgery was conducted by exposing the sciatic nerve in the same manner but without tying the nerve. Hargreaves and von Frey assays were performed with the injured mice 14 days after the surgery.

For a postoperative pain model, an incision pain model was modified according to a previous study [18] (Supplementary Figure S8D). Briefly, mice were anesthetized with 2% isoflurane and hind paws was sterilized with 10% povidone–iodine solution. With a no. 10 scalpel blade, a 5 mm longitudinal incision was made in the glabrous skin and fascia of a hind paw plantar. The underlying muscle was elevated with a sterile curved forcep, with the muscle origin and insertion intact. The incised skin was apposed with two nylon sutures (8-0; Ailee, Korea). Sham mice were not incised or sutured but anesthetized for the same duration.

### 2.2. Open Field Behavioral Tests

The open field was a square opaque white box (40 × 40 × 40 cm) that was divided into a central field (center, 20 × 20 cm) and an outer field (periphery). After drug injection, individual mice were exposed for 30 min to the open field box under dim light, and the total distance traveled, the time spent in the central field, and rearing numbers were monitored with TSE ActiMot/MoTil system (TSE systems, Bad Homburg, Germany) as described previously [19] (Supplementary Figure S8E).

### 2.3. Sticky-Tape Removal Tests

A circular piece of tape, 9 mm in diameter, was placed on the bottom of a hind paw of the animals. The latency of biting or licking to remove the tape was measured as described previously [20] (Supplementary Figure S8B).

### 2.4. Measurements of Food Intake, Water Intake, and Body Weight

Experiments were conducted as described previously [21]. Briefly, mice fasted for 12 h were treated with drugs and then returned to their individual cages. Cumulative food and water consumptions were recorded at 1, 2, 4, and 8 h after drug treatment. For intraperitoneal vehicle, 1% DMSO in sterile saline was used. For long-term measurement, drugs were administered on every third day for forty-eight days and food and water consumptions were recorded at 8 h after every drug treatment. Body weights were measured on every fifth day for fifty days from the first treatment.

### 2.5. Immunostaining Analysis

Lumbar DRG, spinal cord, and glabrous skin biopsies were prepared from mice at 6 weeks. Sections (14 μm for DRG and 30 μm for spinal cord and glabrous skin) prepared using a Leica CM3050s cryotome (Leica Microsystems, Wetzlar, Germany) were permeabilized with 0.2% Triton-X100 for 1 h. For cultured DRG neurons, cultures were fixed in 4% paraformaldehyde for 15 min, followed by permeabilization with 0.1% Triton-X100 for 10 min and blockade with 3% bovine serum albumin in phosphate-buffered saline. Samples were then stained with anti-GPR171 (rabbit, 1:200; Abcam) anti-TRPV1 (mouse, 1:100; Abcam), anti-TRPA1 (sheep, 1:200; Antibodies-online, Aachen, Germany), anti-TRPV4 (sheep, 1:500; Invitrogen, Rockford, IL. USA), anti-CGRP (goat, 1:2000; Abcam), anti-NF200 (mouse, 1:100; Sigma-Aldrich), anti-NeuN (mouse, 1:100; Chemicon, Temecula, CA, USA), and/or anti-PGP9.5 (mouse, 1:50; Abcam) antibodies. After 24 h at 4 °C, sections were stained with secondary antibodies (Alexa 594-labeled or Alexa 488-labeled secondary antibodies; Life Technologies) for 1 h at room temperatures and placed under a coverslip, followed by image acquisition with an iRiS Digital imaging system (Logos Biosystems, Anyang, Korea) or Zeiss LSM 800 confocal microscope (Carl Zeiss, Oberkochen, Germany).

### 2.6. Reverse Transcription–Polymerase Chain Reaction (RT-PCR) and Quantitative RT-PCR

*GPR171* mRNA expression was validated using RT-PCR or qRT-PCR as described previously [22]. We reverse-transcribed 1 μg RNA with the amfiRivert Platinum cDNA synthesis Master Mix (GenDEPOT, Baker, TX, USA). cDNA was subjected to qRT-PCR with the SYBR Green PCR Master Mix (Applied Biosystems, Woolston, UK) or RT-PCR with 5 × PCR Master Mix (Elpisbio, Daejeon, Korea). *β-actin* mRNA was the internal control. Primer sequences are listed below.

For mouse β-Actin: F-GGCTCTTTTCCAGCCTTCCTT, R-CCACCGATCCACACA: GAGTACT; NM_007393

For mouse *GPR171* version 1: F-CTGGCGGTGTCTAATTTGTG, R-TTTCTTCCAG AGGCTTGCTC; NM_173398 

For mouse *GPR171* version 2: F-TGCCGAACATGGTGATTCCC, R-TATGGGACTGACAGCGTG; NM_173398

For mouse *GPR171* version 3: F-GCTTTGTTCCCTACCACGCT, R-TGAG GCGAAAGTCTCGGTG; NM_173398

### 2.7. Cell Cultures and Transfections

Cultures of DRG neurons and HEK293 cells were prepared and transfected as described previously [11,23,24]. All cells were grown at 37 °C and 5% CO_2_ and neurons were used at 48–72 h after culture. For in vivo transfection of small-interfering RNA (siRNA) or scrambled RNA (scRNA), 3 µg of RNA and 5% d-glucose in 7 µg lipofectamine 2000 (Invitrogen) were injected into the spinal cord or peri-sciatic region 24 or 48 h before experiments. Mouse *GPR171*-siRNA (target sequence: sense-CUGUACUAUCAUCUGUCAA, antisense-UUGACAGAUGAUAGUACAG) and a scrambled RNA were purchased from Bioneer Corporation (Daejeon, Korea). cDNA for human *GPR171* was generously gifted by Dr. Jae Young Seong of Korea University.

### 2.8. Fluorescence Intracellular Ca^2+^ Imaging Experiments

The Ca^2+^ imaging experiments were conducted as described previously [25]. Briefly, cells were loaded with 5 μM Fura-2AM dye and 0.02% pluronic F127 for 30 min. The cells were resuspended in 140 mM NaCl, 5 mM KCl, 2 mM CaCl_2_, 1 mM MgCl_2_, 10 mM glucose, and 10 mM HEPES (titrated to pH 7.4 with NaOH). Images of dye-loaded cells were obtained with a cooled charge-coupled device (CCD) camera (Retiga-SRV, Q-imaging Corp., Burnaby, BC, Canada) at a perfusion rate of approximately 4 mL/min of test solutions and at room temperatures. The ratio of fluorescence intensity at 340 nm/380 nm wavelengths in each experiment was analyzed using MetaFluor (Molecular Devices, Sunnyvale, CA, USA).

### 2.9. Spinal Cord Slice Preparation

Mice were decapitated under anesthesia with isoflurane (VetOne, Boise, ID, USA); the lumbar spinal cord was rapidly removed and placed in ice-cold modified artificial cerebrospinal fluid (ACSF) containing 80 mM NaCl, 2.5 mM KCl, 1.25 mM NaH_2_PO_4_, 0.5 mM CaCl_2_, 3.5 mM MgCl_2_, 25 mM NaHCO_3_, 75 mM sucrose, 1.3 mM sodium ascorbate, and 3 mM sodium pyruvate, with pH at 7.4 and osmolality at 310–320 mOsm/kg, bubbled with 95% O_2_ and 5% CO_2_. Spinal cord slices (250–350 μm) were cut horizontally by a VT1200s vibratome (Leica, Germany). The slice was then incubated for approximately 1 h at 33 °C in oxygenated (95% O_2_ and 5% CO_2_) cutting solution containing 125 mM NaCl, 2.5 mM KCl, 2 mM CaCl_2_, 1 mM MgCl_2_, 1.25 mM NaH_2_PO_4_, 26 mM NaHCO_3_, 25 mM D-glucose, 1.3 mM sodium ascorbate, and 3 mM sodium pyruvate, with pH at 7.2 and osmolality at 310–320 mOsm/kg.

### 2.10. Whole-Cell Patch Clamp Recordings

The slices after incubation as described were placed in a recording chamber and perfused with oxygenated recording solution at a rate of 5 mL/min at room temperatures. Whole-cell recording experiments were then performed on lamina 2 dorsal horn neurons. Borosilicate glass pipettes (Sutter Instrument, Novato, CA, USA) with resistance of 3–6 MΩ were filled with internal solution containing 130 mM potassium gluconate, 5 mM KCl, 4 mM Na_2_ATP, 0.5 mM NaGTP, 20 mM HEPES, and 0.5 mM EGTA with pH 7.28 adjusted with KOH and osmolality at 310–320 mOsm/kg. Data were acquired by pClamp 10.0 software (Molecular Devices) with MultiClamp 700B patch-clamp amplifier and Digidata 1550B (Molecular Devices). Data were low-pass filtered at 2 kHz and digitized at 5 kHz.

### 2.11. Ex Vivo Nociceptor Recordings

Ex vivo single-fiber recordings were carried out as previously described [26]. The two-chambered organ bath system (perfusion and recording chambers) was used. The perfusion chamber was continuously superfused with a synthetic interstitial fluid (SIF) composed of 3.5 mmol/L KCl, 107.8 mmol/L NaCl, 0.69 mmol/L MgSO_4_·7H_2_O, 1.53 mmol/Ll CaCl_2_·2H_2_O, 1.67 mmol/L NaH_2_PO_4_·2H_2_O, 26.2 mmol/L NaHCO_3_, 9.64 mmol/L C_6_H_11_NaO_7_, 7.6 mmol/L sucrose, and 5.55 mmol/L glucose, which was saturated with a mixture of 95% O_2_ and 5% CO_2_ and maintained at 31 ± 1 °C. The hairy skin of the hind paw innervated by the saphenous nerve was dissected from mice sacrificed with CO_2_ inhalation. The preparation was put in the perfusion chamber with the epidermal side down. The nerve connected to the skin flap was drawn to the recording chamber filled with paraffin oil. The nerve filaments teased from the nerve were used for single-fiber recordings using gold electrodes. Spikes from single C-fiber nociceptors were recorded with a differential amplifier (DP 311; Warner Instruments, Hamden, CT, USA), and the data were transferred to a personal computer via a data acquisition system (DAP5200a; Microstar Laboratory, Redmond, WA, USA). Data were analyzed using the window discrimination feature of the software (Dapsys 8; Bethel University, http://dapsys.net/ accessed on 5 March 2021) [27].

### 2.12. Compounds

All chemicals were purchased from Sigma-Aldrich (St. Louis, MO, USA) unless otherwise described. BigLEN and MS15203 were purchased from Tocris Bioscience (Ellisville, MO, USA). Cinnamaldehyde was purchased from MP Biomedicals (Solon, OH, USA). Stock solutions were prepared using water or dimethyl sulfoxide and diluted with test solutions before use.

### 2.13. Data Analysis

Statistical significance of data was assessed using the two-tailed unpaired Student’s t-test, one-way analysis of variance (ANOVA) followed by Bonferroni’s post hoc test, or two-way ANOVA followed by Tukey’s post hoc test (*** *p* < 0.001, ** *p* < 0.01, * *p* < 0.05). Data are shown as means ± S.E.M.

### 2.14. Data Availability

All relevant data are available from the authors upon request.

## 3. Results

### 3.1. GPR171 Is Expressed in Nociceptors

**Figure 1:**
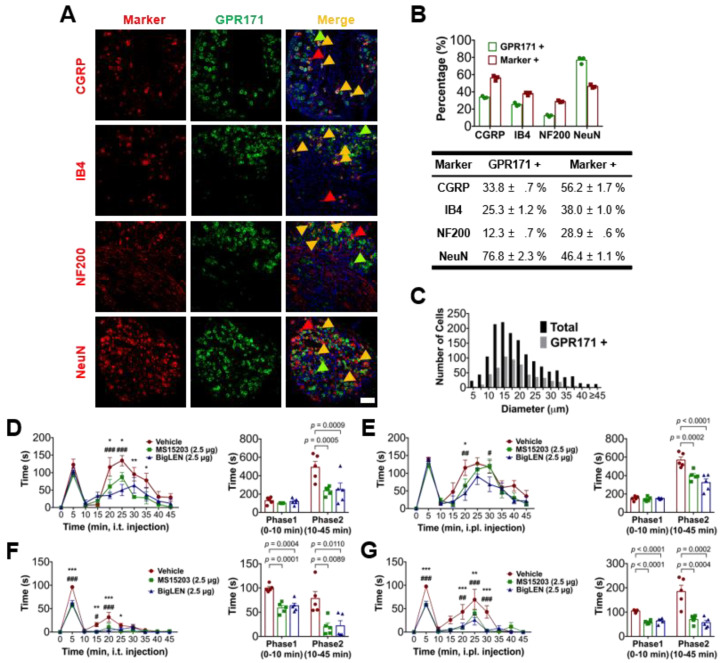
PR171 expression in a subset of dorsal root ganglia (DRG) neurons. (**A**) Double immunostaining of GPR171 with other DRG markers in the lumbar DRG (scale bar, 50 μm). Arrowheads indicate the marker-expressing somas for example. (**B**) Co-expression ratios of GPR171 and other DRG markers. The ratios were calculated as mean percent values by counting the number of GPR171-positive neurons among that of marker-positive neurons from three different mice and vice versa. Data are presented as means ± S.E.M. (**C**) Size distribution of collected DRG neurons and GPR171-positive DRG neurons according to soma diameters. (**D**) Time course of the duration of 5% formalin-induced nociceptive behaviors in mice. Animals were intraplantarily (i.pl.) pretreated with vehicle (red), or GPR171-specific agonists, MS15203 (2.5 μg, green) or bigLEN (2.5 μg, blue), 30 min prior to the formalin injection. The formalin responses were quantified into the two phases of the right histogram. (**E**) Experiments were conducted as in (**D**) but animals were intrathecally (i.t.) pretreated with vehicle or drugs. (**F**) Experiments were conducted as in (**D**) but animals were treated with 0.5% formalin. (G) Experiments were conducted as in (**F**) but animals were intrathecally pretreated with vehicle or drugs. Five animals were used for each data point of (**D**–**G**). Effects on the durations of the nociceptive responses of drugs in (**D**–**G**) at every 5 min (left panels) were compared with those with vehicle injection by two-way ANOVA and Tukey’s test (* for MS15203 treatment and # for bigLEN treatment), and the phase comparisons (right panels) were conducted by one-way ANOVA and Bonferroni’s test. Statistical differences are indicated by * for the effects of MS15203 and # for those of bigLEN, compared to those of the vehicles. *** *p* < 0.001, ** *p* < 0.01, * *p* < 0.05; ^###^ *p* < 0.001, ^##^ *p* < 0.01, ^#^ *p* < 0.05.

We first examined GPR171 immunostaining in DRG, which initiate nociceptive signals. We found GPR171 expression in a subpopulation of DRG neurons (502 of 1498; 33.5%). We further examined whether other subpopulation markers were co-expressed with GPR171 (Figure 1A,B). Peptidergic and non-peptidergic nociceptor markers including calcitonin gene-related peptide (CGRP) and isolectin B4 (IB4)-binding components were co-localized with GPR171 expression as well as the marker for myelinated DRG neurons, neurofilament 200 (NF200). Over fifty percent of the GPR171-positive subpopulation co-expressed CGRP and at least a third of IB4-positive non-peptidergic nociceptors were also GPR171-positive (Figure 1B). Neuronal diameter analysis was also performed on the DRG neurons. Small-diameter neurons, which are mostly comprised of C- and or Aδ-fiber nociceptors, constituted the majority of GPR171-expressing neurons, particularly around neurons with 15–20 μm in diameter (Figure 1C). Approximately half of these small-diameter neurons expressed GPR171, and these proportions were two times greater than the subpopulation with diameters 35 μm and larger. These data suggest that considerable numbers of nociceptor DRG neurons express GPR171. Immunostaining of the glabrous skin layers, spinal cord, and cultured DRG neurons of mice also revealed GPR171 expression (Supplementary Figure S1). DRG nerve termini in the skin epidermis partly showed its co-expression with CGRP but not with IB4 (Supplementary Figure S1A) [28,29]. Thus, these data indicate a potential role of GPR171 in nociceptor DRG neurons.

We then speculated whether GPR171 functions in nociception and examined the effect of its specific activation using the conventional formalin test. Intraplantar and intrathecal administration of endogenous or synthetic GPR171 agonists (bigLEN and MS15203, respectively) both significantly mitigated phase 2 nociceptive behaviors induced by 5% formalin injection (Figure 1D,E). Interestingly, when we performed the Transient receptor potential ankyrin subtype 1 (TRPA1) activation-specific formalin test using a lower dose of formalin (0.5%) [30,31], both phase 1 and 2 nociceptive behaviors were reduced by GPR171 activation (Figure 1F,G). The data suggest that GPR171 expressed in peripheral nociceptors may play a role in modulating their excitability, implicating that modality-specific components such as Transient receptor potential (TRP) ion channels may be involved in this mechanism.

### 3.2. Peripheral GPR171 Activation Attenuates TRP-Specific Acute Pain

**Figure 2:**
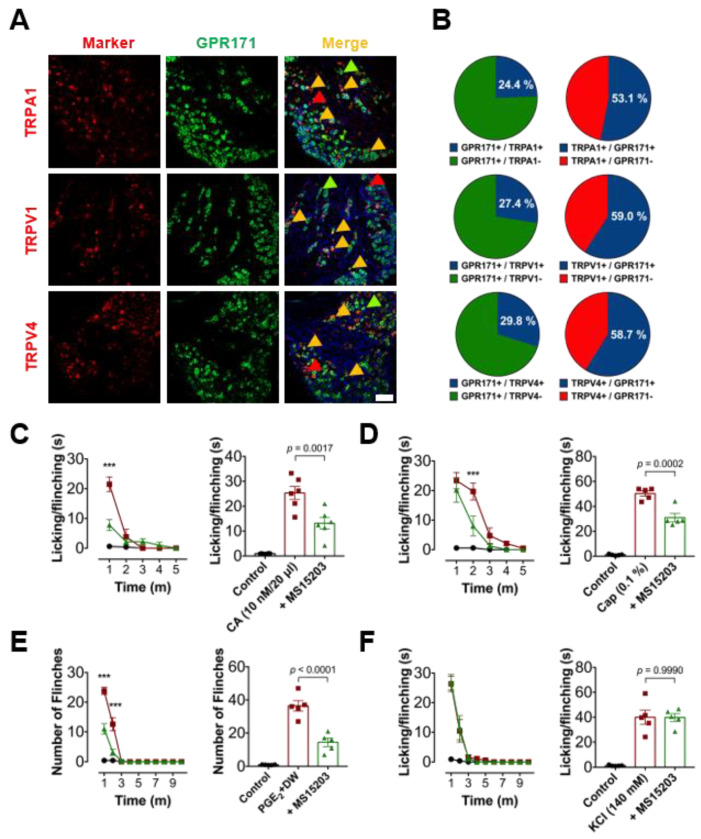
GPR171 expressions in Transient receptor potential (TRP)-expresser neurons and the effect on TRP-mediated nociceptions of GPR171 activation. (**A**) Double immunostaining of GPR171 with TRP channels in the lumbar DRGs (scale bar, 50 μm). (**B**) Co-expression ratios of GPR171 and TRP channels of the neurons from three different mice. Data are presented as means ± S.E.M. (**C**) Time course of the duration of 10 mM cinnamaldehyde (CA)-induced nociceptive behaviors in mice. Animals were intraplantarily pretreated with vehicle (red), or GPR171-specific agonist, MS15203 (2.5 μg, green), 30 min prior to the CA injection. From (**C**) to (**E**), black circles represent the mean data from the animals pretreated with MS150203, 30 min prior to the vehicle injection instead of TRP activators. Total durations of nociceptive responses for 5 min are quantified in the right histogram. (**D**) Time course of the duration of 0.1% capsaicin (CAP)-induced nociceptive behaviors and the effect on it of MS15203 were monitored in the same way as in (**C**). (**E**) Time course of the number of hypotonicity-induced flinches in prostaglandin E2 (PGE2)-primed mice. Animals were intraplantarily pretreated with vehicle (red) or MS15203 (2.5 μg, green), 30 min prior to the hypotonic stimulation. Total number of flinches for 10 min are quantified in the right histogram. (**F**) Time course of the duration of 140 mM KCl-induced nociceptive behaviors in mice. Animals were intraplantarily pretreated with vehicle (red) or MS15203 (2.5 μg, green), 30 min prior to the KCl injection. Total durations of nociceptive responses for 10 min are quantified in the right histogram. In (**C**–**F**), five animals were used for each data. Statistical differences are indicated by * for the effects of MS15203 compared to the responses upon chemical stimulations. *** *p* < 0.001.

Based on the data shown above, we hypothesize possible co-expressions of nociceptive TRP ion channels (TRPV1, TRPA1, and TRPV4). When immunostained, over a half of TRP-positive neurons co-expressed GPR171 and approximately a quarter of the DRG neuronal population expressing GPR171 also expressed at least one of those three TRP channels (Figure 2A,B). Therefore, we then focused on these TRP-specific pain modalities. Licking and flicking induced by intraplantar injection of cinnamaldehyde, which is a TRPA1 activator, and induced by the TRPV1 activator capsaicin as well as flinches in response to TRPV4 activation using hypotonic insult were all blunted when the GPR171 activator, MS15203, was peripherally pre-treated (Figure 2C,E). In contrast, nociceptive behaviors by the activation of voltage-sensing components were not affected (Figure 2F). Thus, rather than general excitability of the nociceptors, TRP-channel-specific mechanisms may be modulated by GPR171 action in these neurons, which may also explain the attenuation of phase 1 nociception because, at least partly, TRPA1 activation is known to be involved.

### 3.3. GPR171 Modulates DRG Neuronal Functions

**Figure 3:**
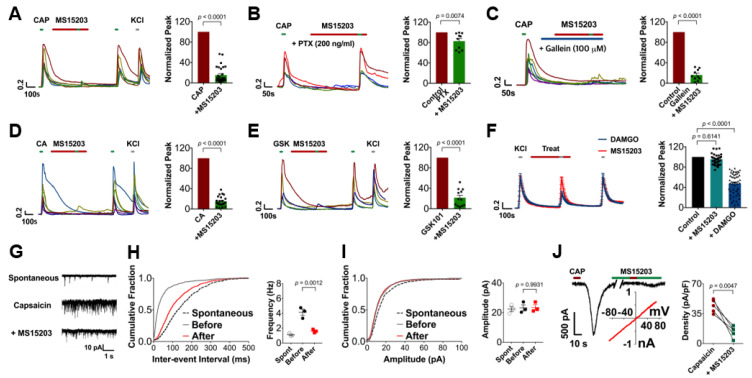
GPR171 modulates TRP channel functions in DRG neurons. (**A**) MS15203 attenuated intracellular Ca^2+^ increases by 0.3 μM capsaicin-induced TRPV1 activation in Fura-2 Ca^2+^ imaging experiments using cultured murine DRG neurons (*n* = 30). Averaged Ca^2+^ peaks are quantified and normalized in the right histogram in (**A**) to (**F**). (**B**) Preincubation of pertussis toxin (PTX) for 18 h prevented the effect of GPR171 agonists shown in (A) (*n* = 10). (**C**) Co-application of 100 µM gallein did not prevent the effect of GPR171 agonists shown in (**A**) (*n* = 10). (**D**) MS15203 attenuated intracellular Ca^2+^ increases by 300 μM cinnamaldehyde-induced TRPA1 activation in Fura-2 Ca^2+^ imaging using cultured DRG neurons (*n* = 23). (**E**) MS15203 attenuated intracellular Ca^2+^ increases by 0.1 μM GSK1016790A (abbreviated to GSK)-induced TRPV4 activation in Fura-2 Ca^2+^ imaging using cultured DRG neurons (*n* = 12). (**F**) The µ-opioid receptor agonist [D-Ala^2^, NMe-Phe^4^, Gly-ol^5^]-enkephalin (DAMGO), but not MS15203, attenuated intracellular Ca^2+^ increases by 60 mM KCl-induced voltage-gated channel activation in Fura-2 Ca^2+^ imaging using cultured DRG neurons (*n* = 36). Experiments shown in (**A**–**F**) were performed in triplicate. KCl stimulation was used for monitoring cell survival at the end of the fluorescence monitoring. (**G**–**I**) Representative traces of capsaicin-induced miniature excitatory postsynaptic currents (mEPSCs) in lamina 2 dorsal horn neurons (**G**) and its cumulative probability plot of the inter-event intervals (**H**) and the peak amplitudes (**I**) of recordings with or without 10 μM MS15203 treatment (*n* = 3). (**J**) Representative traces of capsaicin-induced inward currents in whole-cell voltage clamp recordings of cultured DRG neurons (left) and its quantification of peak amplitudes (right, *n* = 5) with or without 10 μM MS15203 treatment (inset: a current–voltage curve of capsaicin-induced current without MS15203 treatment subtracted by that with MS15203 treatment). The break indicates a 5 min omission on the trace. Data are presented as means ± S.E.M.

We further analyzed the mechanism of GPR171 action using in vitro and ex vivo preparations. In cultured DRG neurons, the TRPV1-mediated increase in intracellular Ca^2+^ upon capsaicin perfusion was blunted by the GPR171-specific agonist, MS15203 or bigLEN, incubation (Figure 3A and Supplementary Figure S3A). This result is reproducible in whole-cell voltage clamp experiments (Figure 3J). The inhibitory effect disappeared when pretreated with pertussis toxin but not with gallein, which confirmed that Gi/o signaling is coupled and that Gα than Gβγ is critical in this GPR action (Figure 3B,C; Supplementary Figure S3A–C) [7]. The inhibition was also observed with TRPA1-, TRPV2-, TRPV4-, TRPM3-, and TRPC4/5-mediated increases in intracellular Ca^2+^ (Figure 3D,E; Supplementary Figure S3D–F). Again, Ca^2+^ responses of DRG neurons by activation of voltage-sensing components that were readily modulable by µ-opioid receptor activation were resistant (Figure 3F), which is confirmed by whole-cell voltage clamp assays (Supplementary Figure S4A–H) and suggests that TRP-channel-specific events were particularly modulable by GPR171 action. Ex vivo C-fiber recording in skin–nerve preparation also showed the same outcomes from peripheral perfusion of bigLEN for inhibition of capsaicin-induced firing (Supplementary Figure S4M,N). Therefore, TRP-mediated excitatory events can be regulated by GPR171 activation in the peripheral termini of nociceptors. We recorded the excitatory post-synaptic current (EPSC) of dorsal horn neurons to observe the effect of GPR171 in the spinal cord. The frequency of EPSC but not its amplitude evoked by capsaicin perfusion was significantly reduced with treatment with either bigLEN or MS15203, indicating that TRPV1-mediated presynaptic events in the DRG central termini were selectively attenuated by GPR171 activation (Figure 3G–I; Supplementary Figure S4I–L). Altogether, these data suggest that TRP activation in nociceptor DRG neurons may be a crucial target of GPR171 action in peripheral nociceptive signaling.

### 3.4. Peripheral GPR171 Activation Attenuates Pathologic Pain

**Figure 4:**
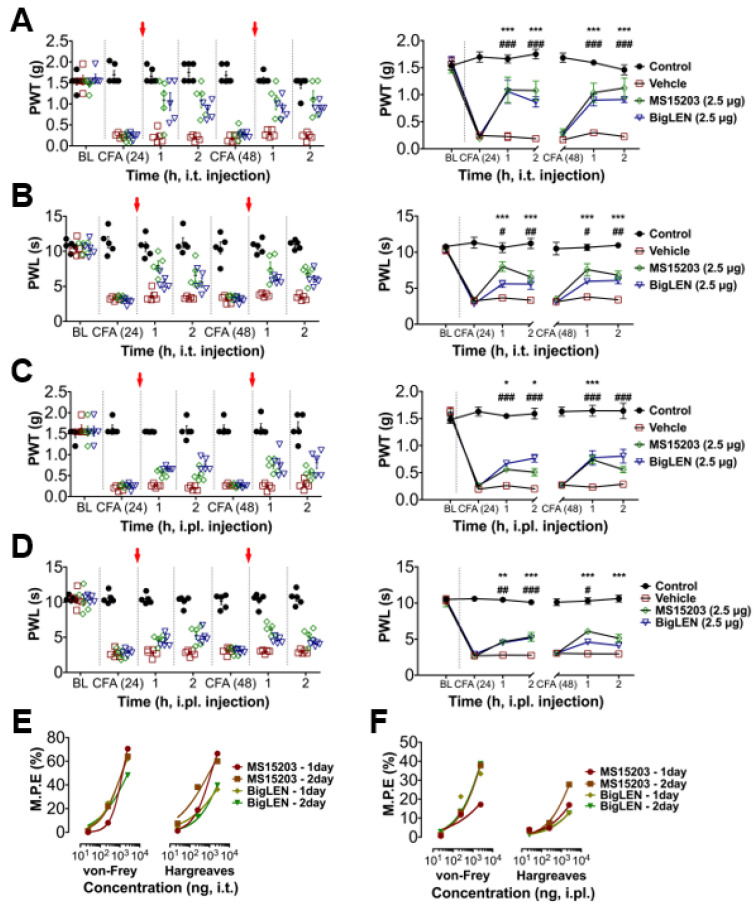
GPR171 activation alleviates inflammatory pain. (**A**) Time course of von Frey paw-withdrawal thresholds (PWTs) in complete Freund’s adjuvant (CFA)-inflamed mice. Animals were intrathecally treated with vehicle (red), MS15203 (2.5 μg, green), or bigLEN (2.5 μg, blue) immediately after 24 and 48 h threshold monitoring. The right line plot shows the mean thresholds. BLs indicate baseline thresholds or latencies under normal condition before inflammation. Red arrows indicate the time for treatments. (**B**) Time course of Hargreaves paw-withdrawal latencies (PWLs) in CFA-inflamed mice. Animals were treated with drugs in the same manner as in (**A**). The right line plot shows the mean latencies. (**C**) Time course of von Frey thresholds in CFA-inflamed mice. Animals were intraplantarily treated with vehicle (red), MS15203 (2.5 μg, green), or bigLEN (2.5 μg, blue) in the ipsilateral hind paws immediately after 24 and 48 h threshold monitoring. The right line plot shows the mean thresholds. (**D**) Time course of Hargreaves latencies in CFA-inflamed mice. Animals were treated with drugs in the same manner as in (**C**). The right line plot shows the mean latencies. Five animals were used for each data point. Statistical significance is represented using different symbols (* for MS15203 treatment and # for bigLEN treatment). (**E**) Maximum possible analgesic effects (percent M.P.E.) were produced from treatments with different doses of GPR171 activators (details are listed in Supplementary Figure S5). Percent M.P.E.s were calculated from comparisons of the subtractions of withdrawal thresholds (or latencies) of the ipsilateral side before and after inflammation with vehicle treatment, to those with drug treatment. Symbols indicate different drug treatments in CFA-inflamed mice (circle, single intrathecal treatment of MS15203 24 h after CFA inflammation; square, double intrathecal treatments of MS15203 24 and 48 h after CFA inflammation; diamond, single intrathecal treatment of bigLEN 24 h after CFA inflammation; inverted triangle, double intrathecal treatments of bigLEN 24 and 48 h after CFA inflammation). (**F**) Percent M.P.E.s were produced from treatments with different doses of GPR171 activators (details are listed in Supplementary Figure S5). Symbols indicate different drug treatments (circle, single intrathecal treatment of MS15203 24 h after CFA inflammation; square, double intrathecal treatments of MS15203 24 and 48 h after CFA inflammation; diamond, single intrathecal treatment of bigLEN 24 h after CFA inflammation; inverted triangle, double intrathecal treatments of bigLEN 24 and 48 h after CFA inflammation). Statistical differences are indicated by * for the effects of MS15203 and # for those of bigLEN, compared to those of the vehicles. *** *p* < 0.001, ** *p* < 0.01, * *p* < 0.05; ^###^ *p* < 0.001, ^##^ *p* < 0.01, ^#^ *p* < 0.05.

**Figure 5:**
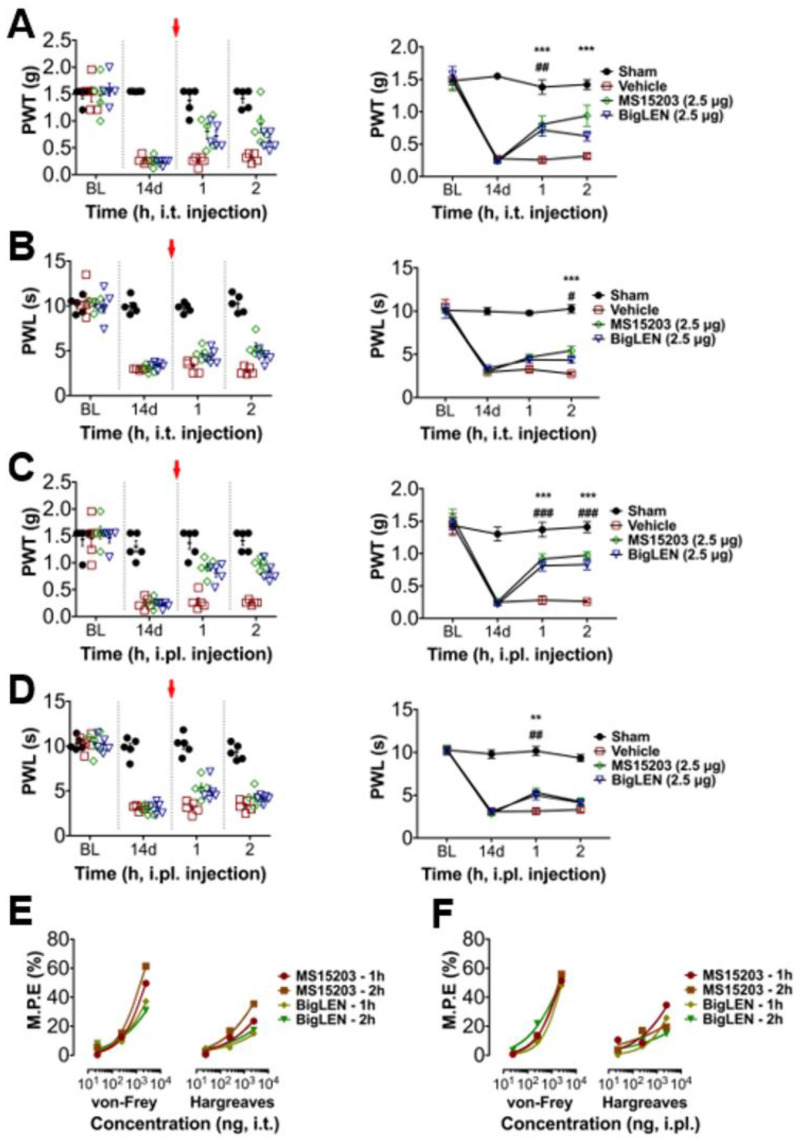
GPR171 activation alleviates neuropathic pain. (**A**) Time course of von Frey paw-withdrawal thresholds in mice with chronic constriction injury (CCI). Animals were intrathecally treated with vehicle (red), MS15203 (2.5 μg, green), or bigLEN (2.5 μg, blue) immediately after 14 days of threshold monitoring. The right line plot shows the mean thresholds. (**B**) Time course of Hargreaves paw-withdrawal latencies in mice with CCI. Animals were treated with drugs in the same manner as in (A). The right line plot shows the mean latencies. (**C**) Time course of von Frey thresholds in mice with chronic constriction injury (CCI). Animals were intraplantarily treated with vehicle (red), MS15203 (2.5 μg, green), or bigLEN (2.5 μg, blue) in the ipsilateral hind paws immediately after 14 days of threshold monitoring. The right line plot shows the mean thresholds. (**D**) Time course of Hargreaves latencies in mice with CCI. Animals were treated with drugs in the same manner as in (**C**). The right line plot shows the mean latencies. Five animals were used for each data point. Statistical significance is represented using different symbols (* for MS15203 treatment and # for bigLEN treatment). (**E**) Percent M.P.E.s were produced from treatments with different doses of GPR171 activators (details are listed in Supplementary Figure S6). Symbols indicate different drug treatments in mice with CCI (circle, 1 h after intrathecal treatment of MS15203; square, 2 h after intrathecal treatment of MS15203; diamond, 1 h after intrathecal treatment of bigLEN; inverted triangle, 2 h after intrathecal treatment of bigLEN). (**F**) Percent M.P.E.s were produced from treatments with different doses of GPR171 activators (details are listed in Supplementary Figure S6). Symbols indicate different drug treatments in mice with CCI (circle, 1 h after intraplantar treatment of MS15203; square, 2 h after intraplantar treatment of MS15203; diamond, 1 h after intraplantar treatment of bigLEN; inverted triangle, 2 h after intraplantar treatment of bigLEN). *** *p* < 0.001, ** *p* < 0.01; ^###^ *p* < 0.001, ^##^ *p* < 0.01.

**Figure 6:**
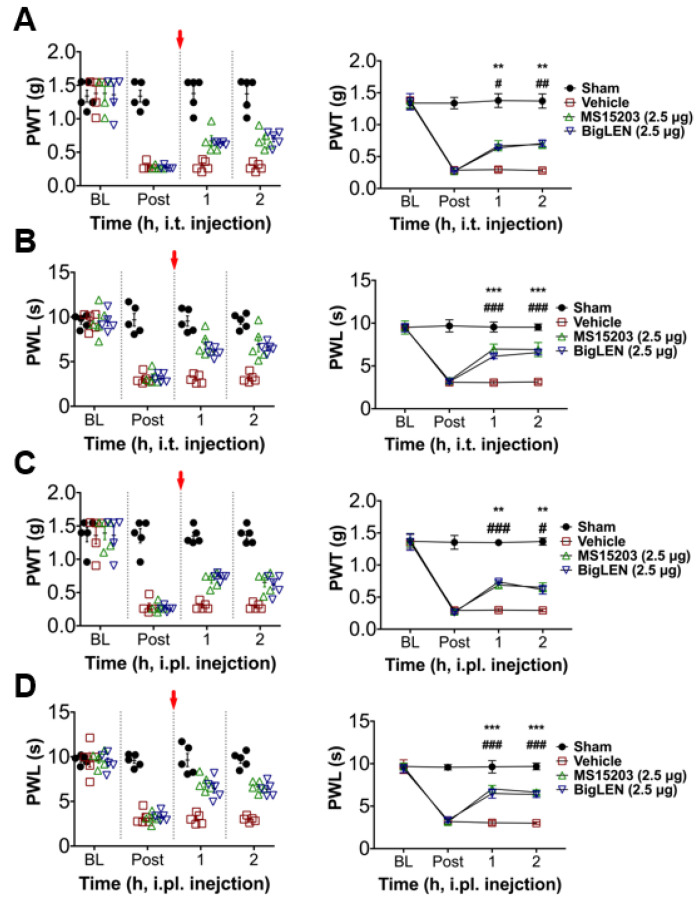
GPR171 activation alleviates incision-induced pain. (**A**) Time course of von Frey thresholds in mice of an incision pain model. Animals were intrathecally treated with vehicle (red), MS15203 (2.5 μg, green), or bigLEN (2.5 μg, blue) immediately after the first post-incision threshold monitoring. Threshold monitoring began immediately after the completion of incision operation. The right line plot shows the mean thresholds. (**B**) Time course of Hargreaves latencies in mice before and after incision. Animals were treated with drugs in the same manner as in (**A**). The right line plot shows the mean latencies. (**C**) Time course of von Frey thresholds in mice before and after incision. Animals were intraplantarily treated with vehicle (red), MS15203 (2.5 μg, green), or bigLEN (2.5 μg, blue) in the ipsilateral hind paws immediately after 24 and 48 h threshold monitoring. The right line plot shows the mean thresholds. (**D**) Time course of Hargreaves latencies in mice before and after incision. Animals were treated with drugs in the same manner as in (**C**). The right line plot shows the mean latencies. In (**A**–**D**), five animals were used for each data point. Statistical significance is represented using different symbols (* for MS15203 treatment and # for bigLEN treatment). *** *p* < 0.001, ** *p* < 0.01; ^###^ *p* < 0.001, ^##^ *p* < 0.01, ^#^ *p* < 0.05.

To determine whether the anti-nociceptive mechanisms of GPR171 work in pathologic pain states in vivo, we first tested peripheral injection of GPR171-specific agonists in complete Freund’s adjuvant (CFA)-induced hind paw inflammatory pain. Two injection routes were selected to examine whether the targeting of peripheral and central termini of nociceptors works. Allodynic mechanical hypersensitivity and hyperalgesic heat hypersensitivity were both improved by intrathecal agonist injection (Figure 4A,B) or by its ipsilateral intraplantar injection (Figure 4C,D). These analgesic effects increased in a dose-dependent manner, which indicates that the analgesia is receptor-dependent and that doses of hundreds nanomolar to micromolar concentrations are relatively effective (Figure 4E,F and Supplementary Figure S5). We also tested the effect of the specific agonists on chronic constriction injury (CCI)-induced pain. The analgesic effects were largely similar but more prominent in mechanical allodynia compared with those observed in CFA-induced pain (Figure 5A–D). These effects also showed dose dependence (Figure 5E,F; Supplementary Figure S6). In an incisional pain model of mice, GRP171 activation was also effective at allodynic and hyperalgesic pain alleviation (Figure 6).

**Figure 7:**
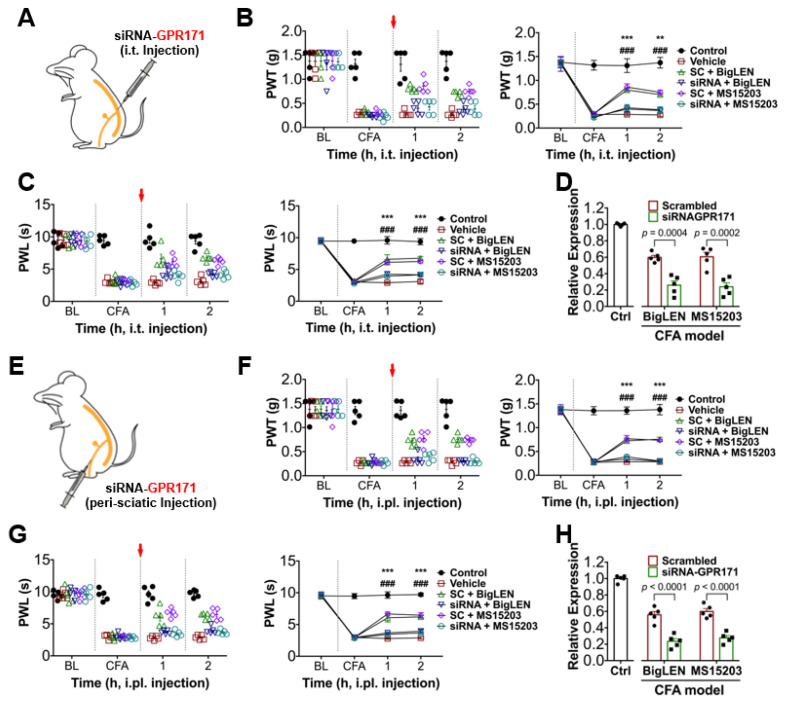
Animals with GPR171 knockdown are tolerant to its pharmacological modulation. (**A**) A cartoon describing the intrathecal (i.t.) treatment of siRNA downregulating GPR171 expression for experiments from (**B**–**D**). Scrambled RNAs (SC) were injected in the same way. (**B**) Time course of von Frey thresholds in complete Freund’s adjuvant (CFA)-inflamed mice. Animals were intrathecally treated with drugs listed in the inset immediately after 24 and 48 h threshold monitoring. The left dot plot shows individual threshold values. (**C**) Time course of Hargreaves latencies in CFA-inflamed mice. Animals were treated with drugs in the same manner as in (**B**). The left dot plot shows individual latency values. (**D**) Expression levels of GPR171 observed by quantitative RT-PCR with lumbar DRGs of the animals intrathecally treated with siRNA compared to those with scrambled RNA. (**E**) A cartoon describing the peri-sciatic nerve treatment of siRNA downregulating GPR171 expression for experiments from (**F**–**H**). Scrambled RNAs were injected in the same way. (**F**) Time course of von Frey thresholds in complete Freund’s adjuvant (CFA)-inflamed mice. Animals were intraplantarily treated with drugs listed in the inset immediately after 24 and 48 h threshold monitoring. The left dot plot shows individual threshold values. (**G**) Time course of Hargreaves latencies in CFA-inflamed mice. Animals were treated with drugs in the same manner as in (**F**). The left dot plot shows individual latency values. (**H**) Expression levels of GPR171 observed by quantitative RT-PCR with lumbar DRGs of the animals peri-sciatically treated with siRNA compared to those with scrambled RNA. *** *p* < 0.001, ** *p* < 0.01; ^###^ *p* < 0.001.

**Figure 8:**
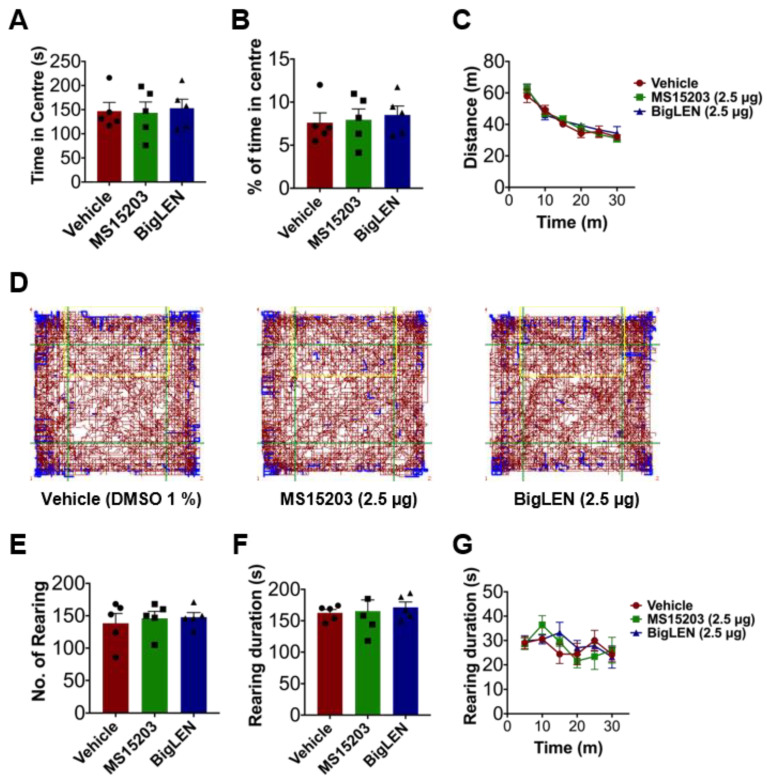
The effect of GPR171 agonist injection on open field behaviors. (**A**) Time spent in the central field of an open field box by mice was monitored for 30 min. Mean seconds ± S.E.M. are shown for five mice with or without intrathecal drug treatment. (**B**) Percent time spent in the central field by mice for 30 min as in (**A**) are shown in the histogram. Mean seconds ± S.E.M. are shown for five mice with or without intrathecal drug treatment. (**C**) Distance traveled with or without intrathecal drug treatment in five mice was averaged during every 5 min period. (**D**) Representative tracks for 30 min of a mouse under open field test are shown (left, vehicle treated; middle, MS15203 treated; right, bigLEN treated). The central field is indicated as a green square. (**E**) Numbers of rearing of mice inside open field box were monitored for 30 min. Mean numbers ± S.E.M. are shown for five mice with or without intrathecal drug treatment. (**F**) Time spent by mice for rearing inside an open field box was monitored for 30 min. Mean seconds ± S.E.M. are shown for five mice with or without intrathecal drug treatment. (**G**) Time spent by five mice for rearing inside an open field box with or without intrathecal drug treatment was averaged every 5 min period. No statistical difference was detected at any data point in one-way ANOVA and Tukey’s analysis.

To determine whether the agonist-induced analgesic effect is GPR171 specific, we conducted RNA interference to downregulate GPR171 expression in the DRG by pre-sciatic or intrathecal injection of its small-interfering RNA and observed the in vivo parameters from the CFA model. Despite bigLEN or MS15203 treatment, pain deteriorated in a similar fashion to those treated with vehicle (Figure 7), indicating that these agonists may specifically act by GPR171 activation. We further determined whether any contribution of psychiatric or motor mechanisms may occur by this GPR activation in the central nervous system, particularly when intrathecally injected, but no such sign was detected in the open field test (Figure 8). We also wondered whether our local intraplantar and intrathecal administrations of GPR modulators may affect eating behaviors, which was previous shown by hypothalamic modulation [21]. We reproduced the mild increase in food intake when intraperitoneally injected, but our local injections did not significantly affect food and water intake and weight gain (Supplementary Figure S2I–M). This tolerance was repeated in mice with GRP171 knockdown (Supplementary Figure S7). We further checked whether innocuous somatosensory functions are affected and showed that those are largely ignorable (Supplementary Figure S2A–H). Overall, our results suggest that the inhibitory actions of GPR171 activation on the nociceptor activity can be extrapolated to the in vivo level with pathologic pain, and thus, GPR171 may be a novel peripheral target for pain modulation.

## 4. Discussion

Our study proposes a new GPR-coupled regulatory mechanism of pain. Use of the neuropeptide ligand, bigLEN, and synthetic ligand, MS15203, has confirmed GPR171 action, as shown in previous studies [7,21]. In peripheral nociceptors, the similar Gi/o-mediated effect of GPR171 activation operates with these two ligands. Either intraplantar injection or intrathecal application of these ligands avoided brain-related behaviors observed in the studies above, suggesting that pain-specific phenotypes can be produced when peripherally modulated.

We did not detect any GPR171 in tissues near the DRG termini, which indicates that GPR171 action in nociceptors rather than non-neuronal actions may be predominant for neuronal modulation. However, the secretion of endogenous ligands for GPR171 including bigLEN and other unknown ones remains as an open question because of the lack of available antibodies. Since neuron-specific expression of Pcsk1n protein, which is the precursor protein for bigLEN cleavage, has been shown, including in embryonic DRG, but not neighboring non-neuronal tissues, paracrine and/or autocrine actions on the nociceptor-secreted bigLEN in some inflamed conditions can be hypothesized [32,33].

In the spinal cord superficial laminae, some neuronal components, but no glial component, expressed GPR171 as shown by immunohistochemical assays. Nonetheless, firing of dorsal horn neurons was only presynaptically modulated by perfusion of GPR171 ligands. Further studies will clarify whether and/or how these GPR171-positive dorsal horn neurons contribute to the modulation of the ascending pain signals. This may also help to fully understand why phase 2 nociception in the formalin test was more consistently affected than that of phase 1 because many other components as well as presynaptic TRPs are known to contribute to phase 2-inducing synaptic sensitization.

DRG is an important painkilling target because DRG-specific modulation may avoid central adverse effects. Neuropeptide-activated and Gi/o-coupled GPRs such as somatostatin and neuropeptide Y receptors, subtypes of which are expressed in a subset of DRG neurons, are currently being revisited in exploring peripheral analgesic efficacy [34,35,36]. Of note, the studies proposed TRP channel inhibition as the downstream effector mechanism. Although Gi/o-coupled signaling may affect the general excitability of DRG neurons, which mainly involves the altered functions of voltage-gated channels or cyclic nucleotide-gated channels, only TRP channel-mediated effects were detected in our study. Thus, the analgesic outcomes observed here may share a common molecular paradigm with previous studies in which peripheral GPR modulation prompts TRP inhibition. Nonetheless, with the current lack of approaches using voltage-dependent subunits, the possibilities need to be carefully monitored to determine whether specific voltage-dependent effectors are influenced in some particular pathological states and locations in the future.

TRP ion channels expressed in nociceptors play a central role in monitoring harmful events in tissues and transforming those into pain signals. In addition to the direct and specific inhibition of each TRP, triggering an inhibitory upstream signal that may commonly attenuate different TRP actions can be an efficient approach for controlling many aspects of pain events. In this context, our results indicate a novel GPR-mediated mechanism that inhibits multiple TRP actions. Our study demonstrated TRPA1, TRPV1, TRPV2, TRPV4, TRPM3, and TRPC4/C5 inhibitions by GPR171 activation. Many of these TRPs have been shown to be engaged in mechanical and thermal nociceptions to some extent, which seems to explain our analgesic phenotypes from the treatment of GRP171 agonists [37]. Such effector-specific outcomes may not be limited to these TRP-mediated ones in this GPR action and other possible important pathologies should be explored in the future.

Very recently, GRP171 is proposed to be present in the periaqueductal gray (PAG) and to regulate opioid signaling there in µ-opioid receptor-dependent fashions [38]. Its activation alone in PAG was not significantly effective, but reinforced µ-receptor-mediated signals, finally enhanced the opioid-induced analgesia. Thus, the outcomes observed here regarding the peripheral mechanisms and those potentially involving the descending inhibitory pathways appear to commonly contribute to pain relief. Further attempts on these analgesic concepts operating in different circuits in more practical ways such as quantitative comparisons in pharmacology and target accessibility may extend the applicable options for promoting the utilities of GPR171 modulators. In conclusion, our results suggest a novel GPR171-mediated pain-relieving mechanism. Such findings may lead to future opportunities to devise novel analgesic strategies.

## Supplementary Materials

The following are available online at https://www.mdpi.com/2227-9059/9/3/256/s1. Figure S1. GPR171 expression in tissues. Figure S2. Effects of treatments of GPR171 activators on acute somatosensations, food intake, water intake, and weight gain. Figure S3. BigLEN modulates DRG neuronal TRP channel functions. Figure S4. BigLEN modulates dorsal horn and DRG neuronal current response upon capsaicin treatment but not voltage-dependent currents in DRG neurons. Figure S5. Administration of GPR171 agonists at various doses alleviates inflammatory pain. Figure S6. Administration of GPR171 agonists at various doses alleviates neuropathic pain. Figure S7. Effects of treatments of GPR171 activators on food intake, water intake, and weight gain in mice with knockdown. Figure S8. Summarized illustrations for animal experiments conducted in this study.
