# Supplementary material for: GPR171 Activation Modulates Nociceptor Functions, Alleviating Pathologic Pain
Source: Biomedicines. 2021 Mar 5;9(3):256. doi: 10.3390/biomedicines9030256 (PMC8001436; doi:10.3390/biomedicines9030256)
Supplement: Supplementary file 1 [file biomedicines-09-00256-s001.pdf]

Supplementary figures and legends

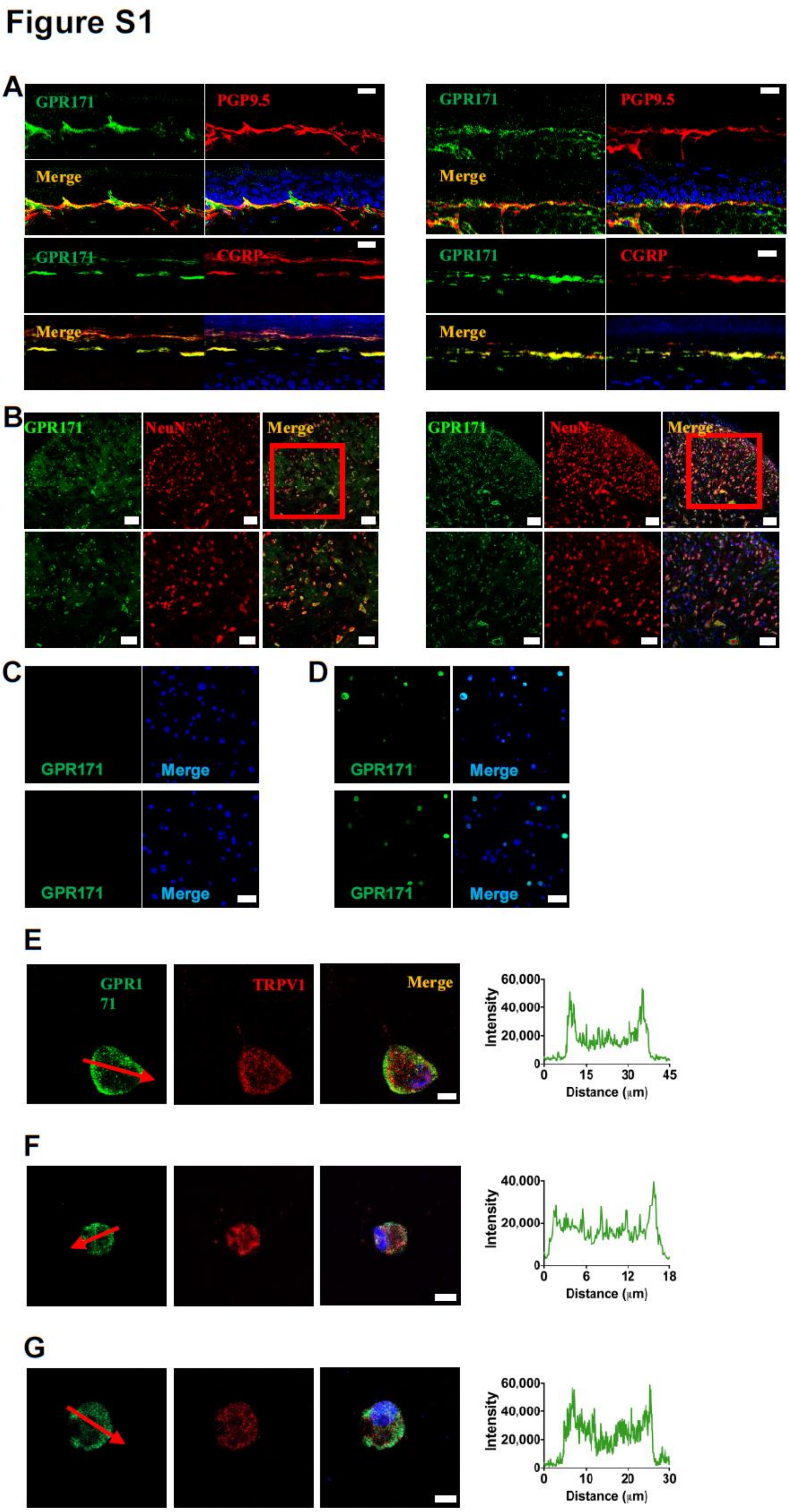

Figure S1. GPR171 expression in tissues.

(A) Immunostaining of GPR171 with a neuronal marker protein gene product 9.5 (PGP 9.5) with or without a pan nuclear marker 4′,6-diamidino-2-phenylindole (DAPI) in the glabrous skin of mice (*top*). Immunostaining of GPR171 with a peptidergic nociceptor marker calcitonin gene-related peptide (CGRP) with or without DAPI in the glabrous skin of mice (*bottom*). Scale bars indicate 20 μm. **(B)** Immunostaining of GPR171 with a neuronal nuclear marker NeuN with or without DAPI in the lumbar spinal cord of mice (scale bar, 50 μm). Presumable glial components indicated by DAPI-positive and NeuN-negative staining did not show GPR171 expression. **(C-D)** Specificity of anti-GPR171 antibody was checked. Immunostaining of GPR171 with or without DAPI in untransfected HEK293 cells (*C*) and those in HEK293 cells transiently transfected with GPR171 (*D*) were shown. Scale bars indicate 50 μm. Experiments shown in *(A) – (D)* were performed in triplicate. **(E-G)** Confocal images of cell bodies of cultured DRG neurons. Regions of interest are marked by red arrows, where fluorescence intensities for GPR171 were quantitated. The data suggest that GPR171 is located in the surface membrane. Scale bars indicate 10 μm.

Figure S2

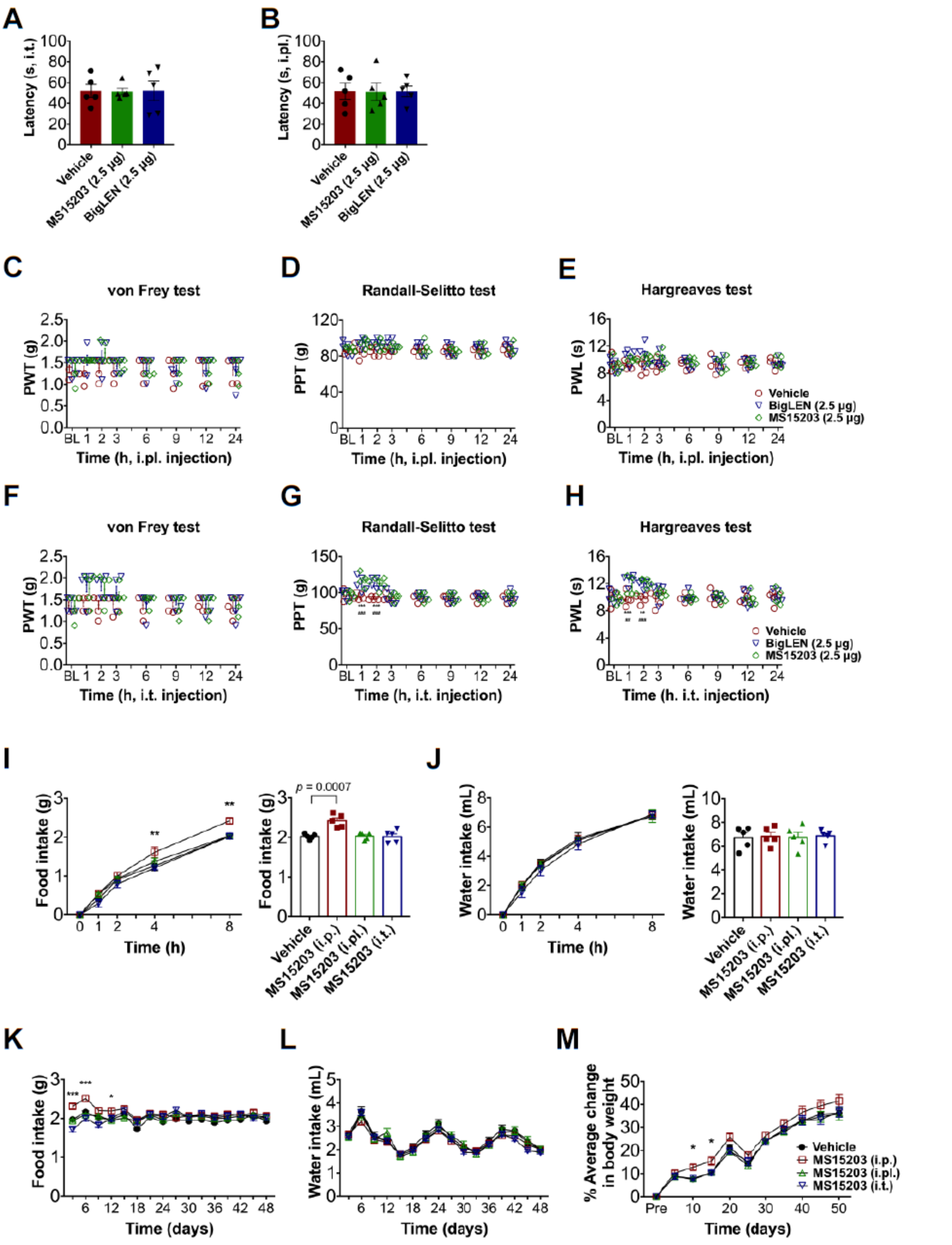

Figure S2. Effects of treatments of GPR171 activators on acute somatosensations, food intake, water intake, and weight gain.

(A) Latencies of removing sticky tape in normal mice. Animals were intrathecally treated with vehicle (red), MS15203 (2.5 μg, green), or bigLEN (2.5 μg, blue). **(B)** Latencies of

removing sticky tape in normal mice. Animals were intraplantarily treated with vehicle (red), MS15203 (2.5 μg, green), or bigLEN (2.5 μg, blue). **(C)** Time course of von Frey thresholds of ipsilateral hind paws in normal mice. Animals were intraplantarily treated with vehicle (red), MS15203 (2.5 μg, green), or bigLEN (2.5 μg, blue). **(D)** Time course of Randall-Selitto thresholds of ipsilateral hind paws in normal mice. Animals were intraplantarily treated in the same manner as in *(C)*. **(E)** Time course of Hargreaves latencies of ipsilateral hind paws in normal mice. Animals were intraplantarily treated in the same manner as in *(C)*. **(F)** Time course of von Frey thresholds of a hind paw in normal mice. Animals were intrathecally treated with vehicle (red), MS15203 (2.5 μg, green), or bigLEN (2.5 μg, blue). **(G)** Time course of Randall-Selitto thresholds of a hind paw in normal mice. Animals were intrathecally treated in the same manner as in *(F)*. **(H)** Time course of Hargreaves latencies of a hind paw in normal mice. Animals were intrathecally treated in the same manner as in *(F)*. **(I)** Cumulative food intake of 12 h fasted mice was recorded for 8 hours after single treatment of drugs. Animals were treated with vehicle (intraperitoneal injection {i.p.}) or MS15203 (3 mg/kg, i.p.; 2.5 μg, i.pl.; 2.5 μg, i.t). Total food consumptions were summarized in the right panel. **(J)** Cumulative water intake was recorded in the same manner as in *(I)*. Animals were treated in the same way as in *(A)*. Total water consumptions were summarized in right panel. **(K)** Eight-hour food intake after drug treatment in the same way as in *(I)* was recorded for forty-eight days. Drugs were treated on every third day. **(L)** Eight-hour water intake after drug treatment in the same manner as in *(I)* was recorded for forty-eight days. Drugs were treated on every third day. **(M)** Body weight changes of animals treated with drugs in *(K)* were recorded on every five day for fifty days. Each data points indicate means ± S.E.M from five animals and their statistic comparisons were conducted by two-tailed unpaired Student's t-test {*(A) – (H)*} or by one-way ANOVA and Bonferroni's {*(I) – (M)*}

Figure S3

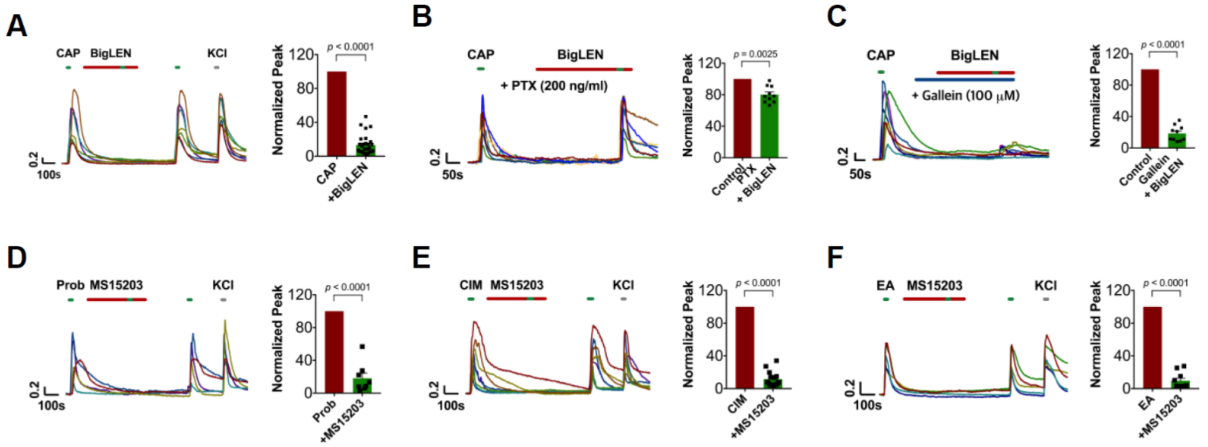

Figure S3. BigLEN modulates DRG neuronal TRP channel functions.

(A) BigLEN attenuated intracellular Ca^{2+} increases by 0.3 μM capsaicin-induced TRPV1 activation in Fura-2 Ca^{2+} imaging experiments using cultured murine DRG neurons (n=29). Averaged Ca^{2+} peaks are quantified and normalized in the right histogram in *(A)* to *(C)*. **(B)** Preincubation of pertussis toxin (PTX) for 18 h prevented the effect of BigLEN shown in *(A)* (n=10). **(C)** Co-application of 100 μM gallein did not prevent the effect of BigLEN shown in *(A)* (n=10). **(D)** MS15203 attenuated intracellular Ca^{2+} increases by 100 μM probenecid (Prob)-induced TRPV2 activation in Fura-2 Ca^{2+} imaging experiments using cultured murine DRG neurons (n=8). Averaged Ca^{2+} peaks are quantified and normalized in the right histogram in *(D)* to *(F)*. **(E)** MS15203 attenuated intracellular Ca^{2+} increases by 1 μM CIM0216 (CIM)-induced TRPM3 activation in Fura-2 Ca^{2+} imaging experiments using cultured murine DRG neurons (n=16). **(F)** MS15203 attenuated intracellular Ca^{2+} increases by 10 μM Englerin A (EA)-induced TRPC4/C5 activation in Fura-2 Ca^{2+} imaging experiments using cultured murine DRG neurons (n=10). Data are presented as means ± S.E.M.

Figure S4

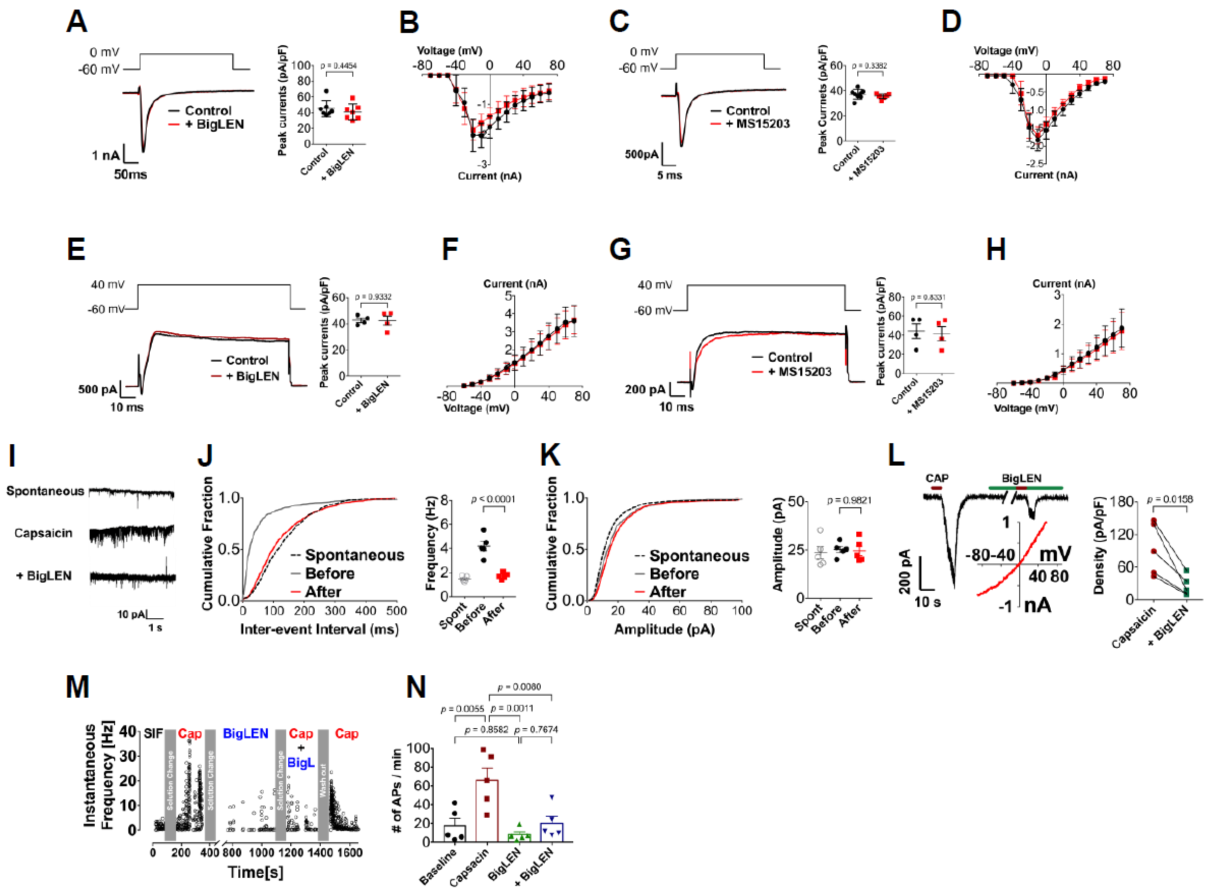

Figure S4. BigLEN modulates dorsal horn and DRG neuronal current response upon capsaicin treatment but not voltage-dependent currents in DRG neurons.

(A) Representative traces of step-depolarization-induced inward currents from a same cultured DRG neurons in whole-cell voltage clamp recordings with or without bigLEN treatment (left). Peak currents at 0 mV step were collected and averaged from the left experiments (right). **(B)** Current-voltage relationships of the peak inward currents from (A) experiments (n=7). **(C)** Representative traces of step-depolarization-induced inward currents from a same cultured DRG neurons in whole-cell voltage clamp recordings with or without MS15203 treatment (left). Peak currents at 0 mV step were collected and averaged from the left experiments (right). **(D)** Current-voltage relationships of the peak inward currents from *(C)* experiments (n=6). **(E)** Representative traces of step-depolarization-induced outward currents from a same cultured DRG neurons in whole-cell voltage clamp recordings with or

without BigLEN treatment (left). Peak currents at 0 mV step were collected and averaged from the left experiments (right). **(F)** Current-voltage relationships of the peak outward currents from *(E)* experiments (n=4). **(G)** Representative traces of step-depolarization-induced outward currents from a same cultured DRG neurons in whole-cell voltage clamp recordings with or without MS15203 treatment (left). Peak currents at 0 mV step were collected and averaged from the left experiments (right). **(H)** Current-voltage relationships of the peak outward currents from *(G)* experiments (n=4). **(I-K)** Representative traces of capsaicin-induced miniature excitatory postsynaptic currents (mEPSCs) in lamina 2 dorsal horn neurons *(I)* and its cumulative probability plot of the inter-event intervals *(J)* and the peak amplitudes *(K)* of recordings with or without 1 μM bigLEN treatment (n=5). **(L)** Representative traces of capsaicin-induced inward currents in whole-cell voltage clamp recordings of cultured DRG neurons (left) and its quantification of peak amplitudes (right) with or without 1 μM bigLEN treatment (inset: a current-voltage curve of capsaicin-induced current without bigLEN treatment subtracted by that with bigLEN treatment) (n=5). The break indicates 5- minute omission on the trace. Data points indicate means ± S.E.M and their statistic comparisons were conducted by two-tailed unpaired Student's t-test {*(A) – (L)*}. **(M)** Representative traces of action potential firing in *ex vivo* nerve fiber recording in skin flap-saphenous nerve preparations. Upon treatment with 100 μM bigLEN, 1 μM capsaicin (abbreviated to Cap)-induced increases in action potential firing of C-fibers were blunted. After bigLEN was washed out, capsaicin responses were recovered. **(N)** The experiments shown in *(M)* were repeated with five different C-fibers from three mice and the data were statistically analyzed by one-way ANOVA and Bonferroni's test.

Figure S5

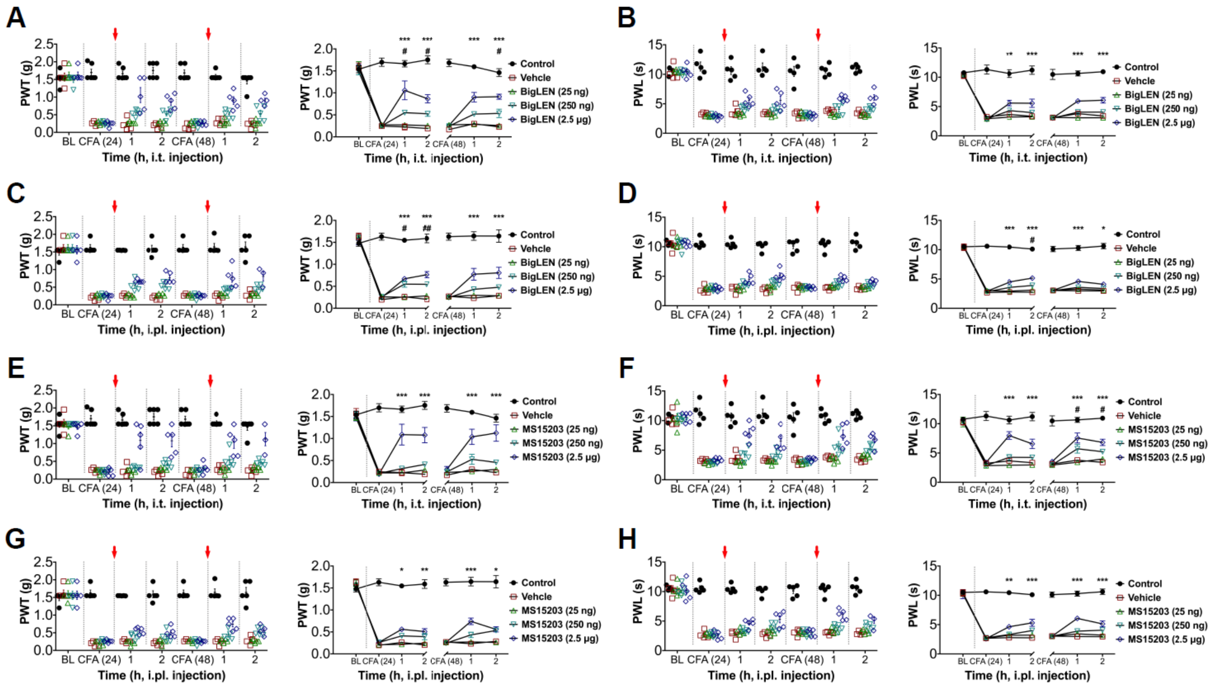

Figure S5. Administration of GPR171 agonists at various doses alleviates inflammatory pain.

(A) Time course of von Frey thresholds in CFA-inflamed mice. Animals were intrathecally treated with vehicle (red) or three different doses of MS15203 (25 ng, green; 250 ng, cyan, 2.5 µg, blue) immediately after 24 h and 48 h threshold monitoring. The right line plot shows the mean thresholds. **(B)** Time course of Hargreaves latencies in CFA-inflamed mice. Animals were treated with drugs in the same manner as in *(A)*. The right line plot shows the mean latencies. **(C)** Time course of von Frey thresholds in CFA-inflamed mice. Animals were intraplantarily treated with vehicle (red) or three different doses of MS15203 (25 ng, green; 250 ng, cyan, 2.5 µg, blue) in the ipsilateral hind paws immediately after 24 h and 48 h threshold monitoring. The right line plot shows the mean thresholds. **(D)** Time course of Hargreaves latencies in CFA-inflamed mice. Animals were treated with drugs in the same manner as in *(C)*. The right line plot shows the mean latencies. **(E)** Time course of von Frey thresholds in CFA-inflamed mice. Animals were intrathecally treated with vehicle (red) or three different doses of bigLEN (25 ng, green; 250 ng, cyan, 2.5 µg, blue) immediately after

24 h and 48 h threshold monitoring. The right line plot shows the mean thresholds. **(F)** Time course of Hargreaves latencies in CFA-inflamed mice. Animals were treated with drugs in the same manner as in *(E)*. The right line plot shows the mean latencies. **(G)** Time course of von Frey thresholds in CFA-inflamed mice. Animals were intraplantarily treated with vehicle (red) or three different doses of bigLEN (25 ng, green; 250 ng, cyan, 2.5 μg, blue) in the ipsilateral hind paws immediately after 24 h and 48 h threshold monitoring. The right line plot shows the mean thresholds. **(H)** Time course of Hargreaves latencies in CFA-inflamed mice. Animals were treated with drugs in the same manner as in *(G)*. The right line plot shows the mean latencies. In *(A-H)*, five animals were used for each data point.

Figure S6

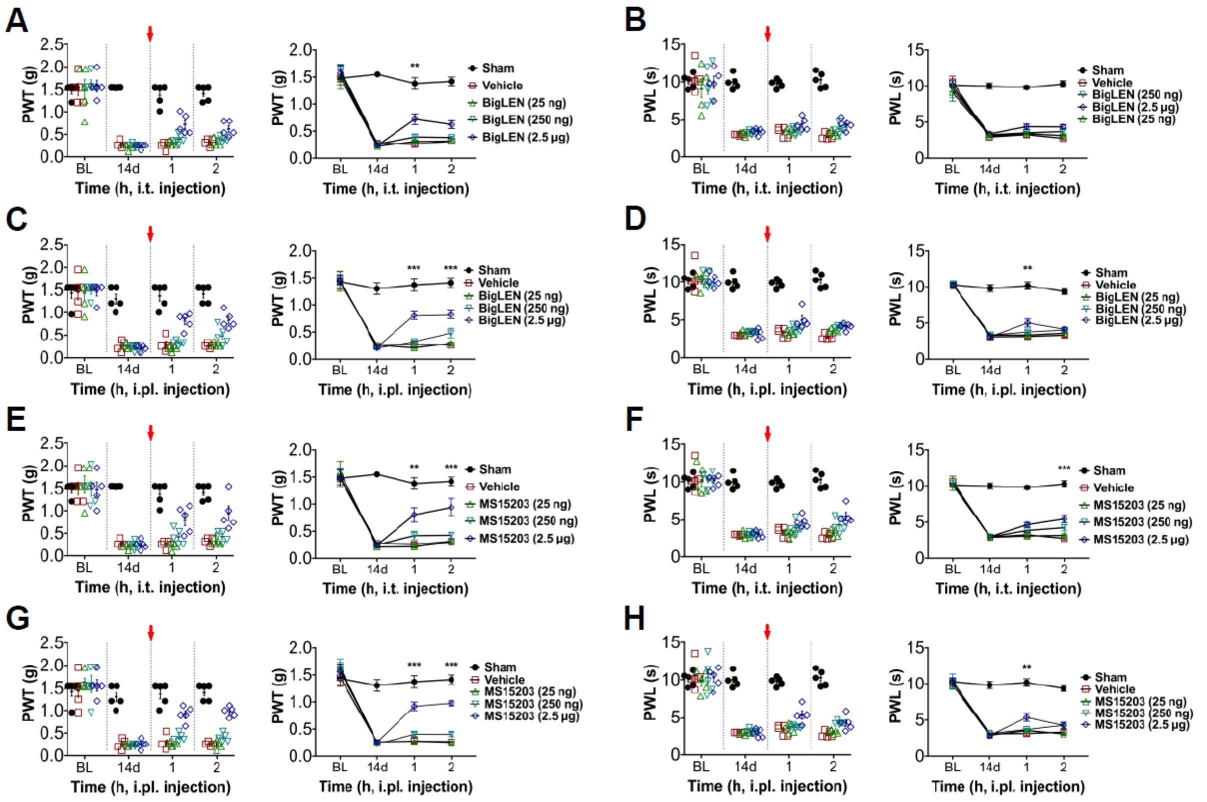

Figure S6. Administration of GPR171 agonists at various doses alleviates neuropathic pain.

(A) Time course of von Frey thresholds in mice with CCI. Animals were intrathecally treated with vehicle (red) or three different doses of MS15203 (25 ng, green; 250 ng, cyan, 2.5 μg, blue) immediately after 14 d threshold monitoring. The right line plot shows the mean thresholds. **(B)** Time course of Hargreaves latencies in mice with CCI. Animals were treated with drugs in the same manner as in *(A)*. The right line plot shows the mean latencies. **(C)** Time course of von Frey thresholds in mice with CCI. Animals were intraplantarily treated with vehicle (red) or three different doses of MS15203 (25 ng, green; 250 ng, cyan, 2.5 μg, blue) in the ipsilateral hind paws immediately after 14 d threshold monitoring. The right line plot shows the mean thresholds. **(D)** Time course of Hargreaves latencies in mice with CCI. Animals were treated with drugs in the same manner as in *(C)*. The right line plot shows the mean latencies. **(E)** Time course of von Frey thresholds in mice with CCI. Animals were

intrathecally treated with vehicle (red) or three different doses of bigLEN (25 ng, green; 250 ng, cyan, 2.5 μg, blue) immediately after 14 d threshold monitoring. The right line plot shows the mean thresholds. **(F)** Time course of Hargreaves latencies in mice with CCI. Animals were treated with drugs in the same manner as in *(E)*. The right line plot shows the mean latencies. **(G)** Time course of von Frey thresholds in mice with CCI. Animals were intraplantarily treated with vehicle (red) or three different doses of bigLEN (25 ng, green; 250 ng, cyan, 2.5 μg, blue) in the ipsilateral hind paws immediately after 14 d threshold monitoring. The right line plot shows the mean thresholds. **(H)** Time course of Hargreaves latencies in mice with CCI. Animals were treated with drugs in the same manner as in *(G)*. The right line plot shows the mean latencies. In *(A-H)*, five animals were used for each data point.

Figure S7

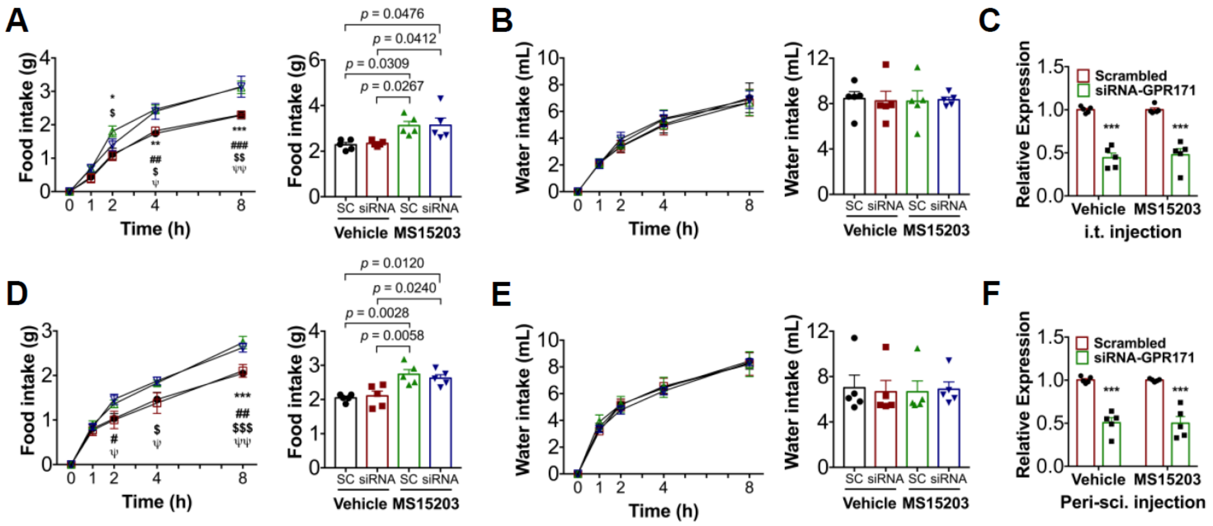

Figure S7. Effects of treatments of GPR171 activators on food intake, water intake, and weight gain in mice with knockdown

Mice treated as in figure 7A for intrathecal RNA injection were used. **(A)** Cumulative food intake of 12 h fasted mice was recorded for 8 hours after single intraperitoneal treatment of MS15203 (3 mg/kg). Total food consumptions were summarized in the right panel. **(B)** Cumulative water intake was recorded in the same manner as in *(A)*. Animals were treated in the same way as in *(A)*. Total water consumptions were summarized in right panel. **(C)** Expression levels of *GPR171* in animals treated with siRNA compared to those with scrambled RNA in experiments *(A)* and *(B)*. **(D)** Mice treated as in figure 7E for peri-sciatic RNA injection were used. Cumulative food intake of 12 h fasted mice was recorded for 8 hours after single intraperitoneal treatment of MS15203 (3 mg/kg). Total food consumptions were summarized in the right panel. **(E)** Cumulative water intake was recorded in the same manner as in *(D)*. Animals were treated in the same way as in *(D)*. Total water consumptions were summarized in right panel. **(F)** Expression levels of *GPR171* in animals treated with siRNA compared to those with scrambled RNA in experiments *(D)* and *(E)*. Each data points indicate means ± S.E.M from five animals and their statistic comparisons were conducted by one-way ANOVA and Bonferroni’s test (* for SC group treated with MS15203; # for siRNA

group treated with MS15203; $ for SC group treated with vehicle; Ψ siRNA group treated with vehicle).

Figure S8

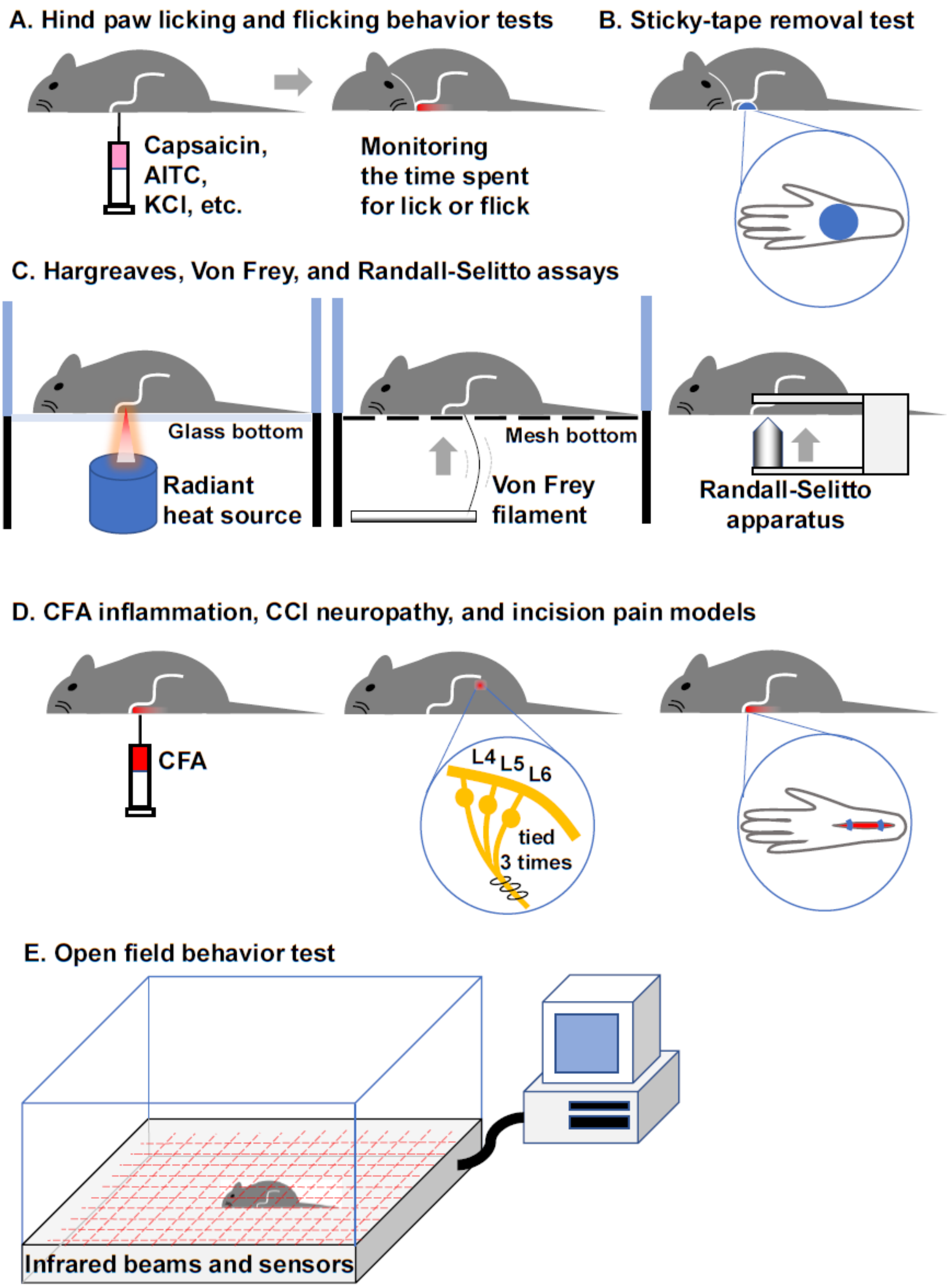

Figure S8. Summarized illustrations for animal experiments conducted in this study.(A) hind paw acute pain tests. **(B)** Sticky-tape removal test. **(C)** Hargreaves, Von Frey, and Randall-Selitto assays. **(D)** Complete Freund's adjuvant (CFA)–induced inflammation model, chronic constriction injury (CCI) model, and incision pain model. **(E)** Open field behavior test.
